# Supplementary figures and images for: Testing the feasibility and utility of an executive function battery for use with primary school-aged students in Malawi
Source: PLOS Glob Public Health. 2025 Jul 11;5(7):e0004680. doi: 10.1371/journal.pgph.0004680 (PMC12250566; doi:10.1371/journal.pgph.0004680)

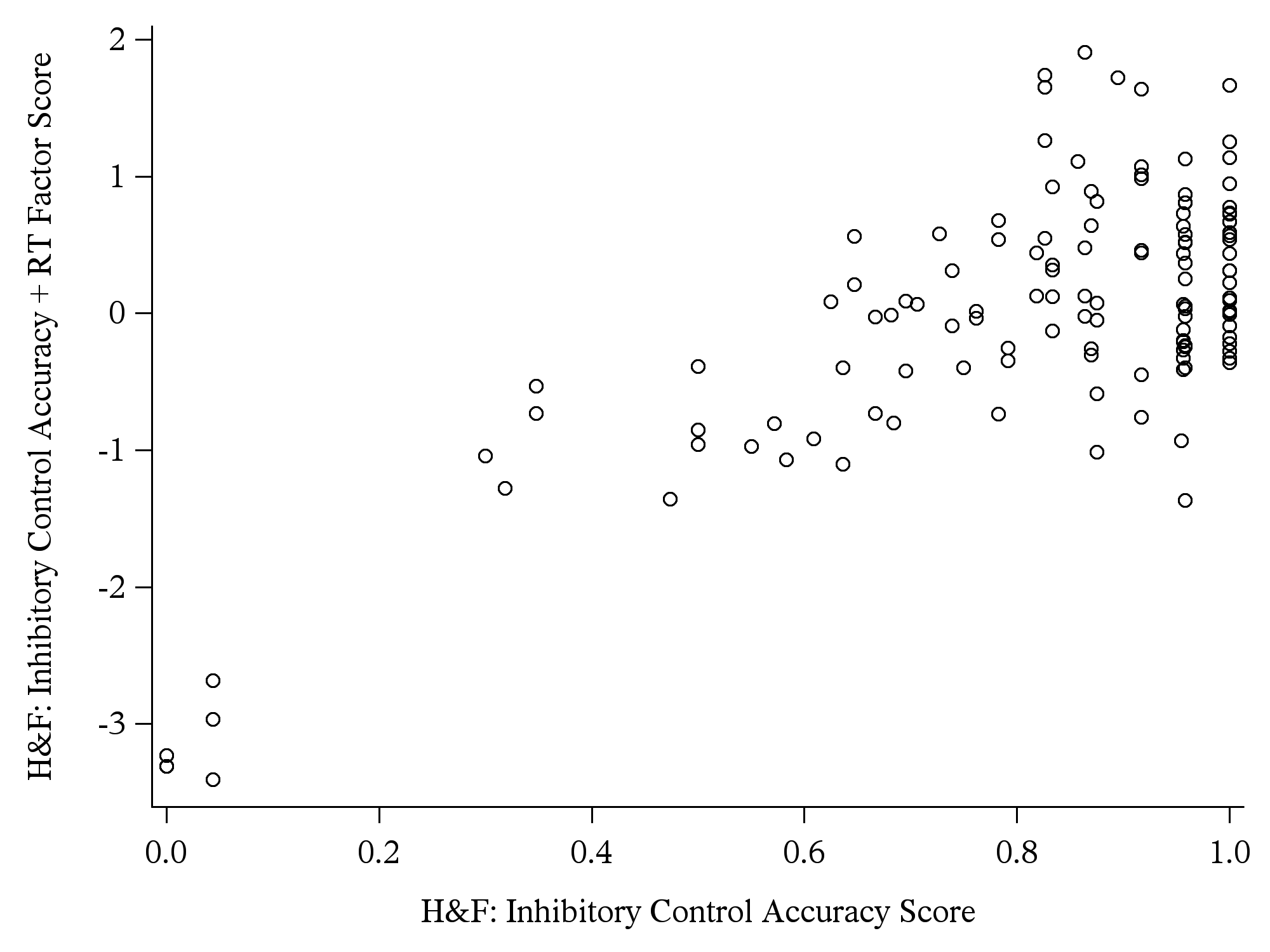

Supplement: S1 Fig — Factor scores that combine item-level information on accuracy and reaction time (y-axis) exhibit improved variation relative to accuracy only (x-axis) scores. (TIF) [file pgph.0004680.s001.tif]

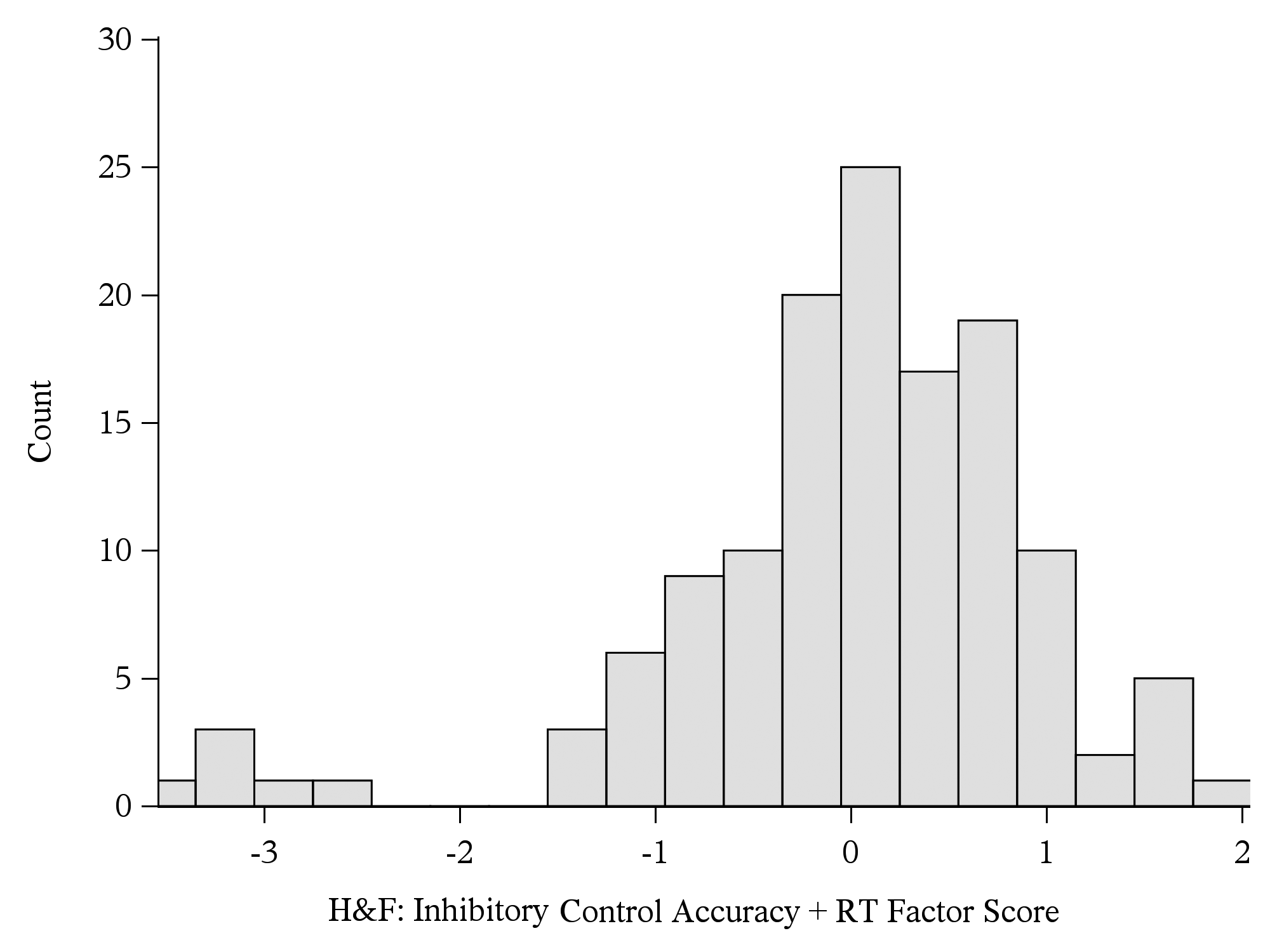

Supplement: S2 Fig — The univariate distribution of combined accuracy + reaction time factor score preserves individual differences in task performance, even among students who complete 100% of items correctly. (TIF) [file pgph.0004680.s002.tif]

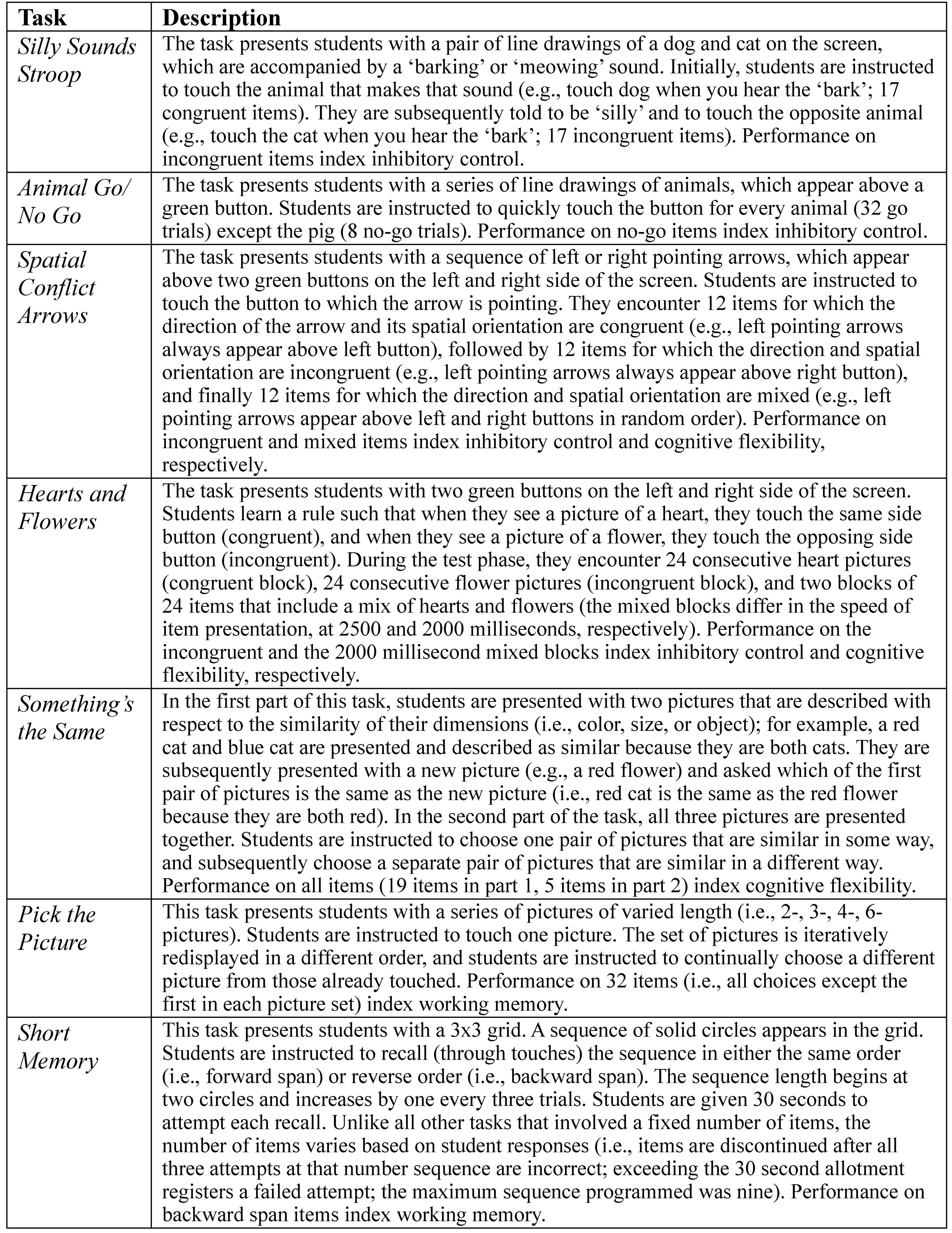

Supplement: S1 Table — (TIF) [file pgph.0004680.s004.tif]
