# Supplementary material for: Testing the feasibility and utility of an executive function battery for use with primary school-aged students in Malawi
Source: PLOS Glob Public Health. 2025 Jul 11;5(7):e0004680. doi: 10.1371/journal.pgph.0004680 (PMC12250566; doi:10.1371/journal.pgph.0004680)
Supplement: Attachment — Submitted filename: RespReview_1.docx [file pgph.0004680.s006.docx]

Dr. Shin,

Below, we summarize our responses to your and Reviewer comments. Below, all references to line numbers refer to the tracked changes version (i.e., RevisedManuscript_TrackChange.docx) of the manuscript. To avoid confusion, we removed line numbers from the uploaded clean version of the manuscript.

Sincerely,

Michael Willoughby, Ph.D.

Senior Fellow, Early Childhood Education

Journal Requirements/Please include a complete copy of PLOS’ questionnaire on inclusivity in global research in your revised manuscript.

We have included the requested questionnaire in our supplemental material and referenced this material in our Methods.

Journal Requirements/Your current Financial Disclosure states, “This study was conducted using internal funds from RTI International.”. However, your funding information on the submission form indicates that you did not receive funding. Please indicate by return email the full and correct funding information for your study and confirm the order in which funding contributions should appear. Please be sure to indicate whether the funders played any role in the study design, data collection and analysis, decision to publish, or preparation of the manuscript.

We apologize for the confusion. We did not receive any *external* funding for this work. RTI International provided *internal* institutional research and development funding to Dr. Reithinger to conduct this work. Per the Editor instruction, we clarified in an email that the award of internal funds did not result in any requirements or advice related to study design, data collection and analysis, the decision to publish, or the preparation of this manuscript.

Journal Requirements/We do not publish any copyright or trademark symbols that usually accompany proprietary names, eg (R), (C), or TM  (e.g. next to drug or reagent names). Please remove all instances of trademark/copyright symbols throughout the text, including TM on page 5 and in abstract.

We removed trademark symbol (™) when referring to Tangerine software in the text (Abstract and **line 133**).

Journal Requirements/In the online submission form, you indicated that Access to study data may be made available upon reasonable request to the contact author and at the discretion of the ethics review committee. All PLOS journals now require all data underlying the findings described in their manuscript to be freely available to other researchers, either: 1. In a public repository; 2. Within the manuscript itself; or, 3. Uploaded as supplementary information. This policy applies to all data except where public deposition would breach compliance with the protocol approved by your research ethics board.

We have been in contact with the chairman of Kamuzu University’s College of Medicine Research Ethics Committee, who granted us permission to make deidentified data available in supplementary materials. We indicated this in the online submission form and the revised manuscript (**lines 152-153**).

Reviewer #1/In this paper by Willoughby et al, assessed the feasibility of a tablet-based tool to assess executive function in Malawian children from grades 1 to 4. This is an interesting question that aims to address challenges in assessing developmental outcomes in children that don’t require hours of testing by highly specialized test administrators. The ability to create a game-based platform that is engaging and provides reliable information on cognition and executive function would be transformative. In this paper, the authors demonstrate that it is easy to assess many children within a short period in school-based settings using this tool. I have some concerns about the construct validity of this tool, and the relatively weak correlation between the tasks meant to reflect the same sub-domain. Additional studies are needed to evaluate the correlation between these executive function tasks and reference tools to demonstrate that the construct validity is retained, particularly when modifications are made.

We appreciate the overall positive disposition towards our study. Based on the Reviewer’s comments, we revised the manuscript (**lines 43-79**) to clarify the following:

1. We have been iteratively developing this battery of tasks for over a decade—initially in the United States and more recently in Kenya and now Malawi.
2. *All* tasks make use of well-established neuropsychological paradigms for measuring subdomains of executive function (EF). That is, we are not developing new tasks. Instead, we are providing a game-like context for presenting existing EF paradigms for use with children. For example, our *Pick the Picture* task makes use of a self-ordered pointing paradigm that has been in use for over four decades (i.e., Petrides & Milner, B. [1982]. Deficits on subject-ordered tasks after frontal- and temporal-lobe lesions in man. *Neuropsychologia, 20*(3), 249-262).
3. We have previously reported on the psychometric properties and construct validity of the *preschool* (preprimary) version of this battery of tasks.
4. The primary objective of this study is to consider the feasibility and initial promise of an expanded *school-aged* (primary) version of these same tasks.
5. Given the promise that was established here, we fully acknowledge that subsequent studies should investigate the psychometric properties and construct validity of these age-expanded tasks (**lines 264-270**).

The Reviewer astutely noted that that EF tasks that purportedly measure the same subdomain were only modestly correlated with each other. This phenomenon has been consistently observed in literature; it is not unique to our battery or this sample. Elsewhere, we have elaborated the implications of this phenomenon as it applies to the measurement of EF skills (including advocating for the use of composite scores, not latent variables, to summarize task performance). Because this idea is tangential to our focal questions, it was not discussed in the manuscript. However, the Reviewer may find these papers of interest:

Camerota, M., Willoughby, M.T., & Blair, C.B. (2020). Measurement models for studying child executive functioning: Questioning the status quo. *Developmental Psychology, 56* (12), 2236-2245. DOI: 10.1037/dev0001127; <https://www.ncbi.nlm.nih.gov/pubmed/33104374>

Willoughby, M.T., Blair, C.B., and the Family Life Project Investigators (2016). Measuring executive function in early childhood: A case for formative measurement. *Psychological Assessment, 28*(3), 319-30. DOI: 10.1037/pas0000152. <https://www.ncbi.nlm.nih.gov/pubmed/26121388>

Willoughby, M.T., Holochwost, S.J., Blanton, Z.E., & Blair, C.B. (2014). Executive Functions: Formative Versus Reflective Measurement. *Measurement: Interdisciplinary Research and Perspectives, 12*(3), 69-95. DOI: 10.1080/15366367.2014.929453

Reviewer #1/The authors note that the goal of the work is to assess the feasibility of implementing a tablet-based test to assess executive function. I feel like they have demonstrated feasibility. However, there is a bigger question of whether this test is a valid assessment of executive function and the domains assessed and whether they retain their construct validity in the translation and in this population unaccustomed to touch-screen technology. This is a bigger question that is not addressed or even mentioned and seems like a significant oversight. Just because we can administer the test and get results that demonstrate reasonable dispersion and children are able to complete the tasks doesn't mean they are accurately measuring the domains of interest.

We appreciate that the Reviewer agrees that we have established the feasibility and initial promise of the age-expanded versions of our EF task battery—this was the primary objective of this study.

As described above, we revised the text to highlight prior evidence of the psychometric properties and validity of our EF task battery that involved preschool-aged versions of these tasks (**lines 43-58**).

We also added text that clarifies the growing use of touch-screen technology for cognitive assessments in the Global South (reviewed by McHenry and colleagues, see **lines 60-61**).

Finally, we added text to the Discussion describing that validation of these age-expanded tasks is an important direction for future research (**lines 264-271**).

Reviewer #1/The authors generated the executive function composite by taking the mean of the tasks completed. Six tasks were included that represented inhibitory control, cognitive flexibility, and working memory. However, not all tasks were completed by all children. To generate a balanced score reflecting these three domains, the mean per domain should be calculated and then averaged to develop an overall composite.

Thank you for this suggestion. We re-scored the composite as suggested and updated the results (**lines 189-201; Table 1; Figure 3**). This alternative scoring approach did not result in any substantive changes to our conclusions from the initial submission.

Reviewer #1/In the challenges of executive function assessment, the authors note student behavioral challenges, including poor concentration, fatigue, and developmental concerns. Ideally, these assessments should be administered in a quiet environment where children are well-rested and can concentrate. Details on test administration (e.g., time of day) and the ability to take breaks are not provided.

We revised the text to clarify that we conducted 1:1 testing with students, provided opportunities for students to take breaks in between tasks (based on student request or assessor suggestion), and that all testing occurred during the typical school day (**lines 145-147**). The modular nature of the task battery (i.e., most tasks take ~5 minutes/each to complete) facilitates the opportunity for breaks. In our experience, it is not uncommon (and indeed expected) for some students to exhibit lapses in attention or motivation during testing.

Reviewer #1/In the Discussion, the authors recommend modifications to the battery of executive function tasks to drop certain games based on the experiences noted and scores generated. However, there is no discussion about the impact of modifications on the validity of the tests or the need to ensure they are measuring what they say they are before implementation of the tool.

The primary goal of this task was to evaluate the success of age-expanded versions of existing EF tasks. Our results indicate that some tasks performed poorly (e.g., comprehension problems for Arrows; ceiling effect for Animal Go/No Go). Identifying these problems in this study will improve the efficiency of future studies (i.e., it makes no sense to consider the validity of these tasks if there are likely to be comprehension or distributional problems). We administer multiple tasks per construct to offset problems with individual tasks (e.g., Hearts & Flowers and Arrows tasks make similar cognitive demands and measure similar phenomena, but the former was clearly better understood by students than the latter).
